# Retraction: Functional Characterization of Two Structurally Novel Diacylglycerol Acyltransferase2 Isozymes Responsible for the Enhanced Production of Stearate-Rich Storage Lipid in *Candida tropicalis* SY005

**DOI:** 10.1371/journal.pone.0187146

**Published:** 2017-10-23

**Authors:** The PLOS ONE Editors

The authors and editors retract this publication [1] following an investigation into concerns around the images presented in several figures. After the publication of this article, readers raised concerns about potential image duplications in Figures 4A-C, 5A-B, 6C, 7A, and 7C. Specifically,

- There are similarities between gel bands in Figure 4C and Figure 7B.
- The backgrounds and first two lanes of Figures 4A and 7C appear to be identical.
- Figure 4B: there are areas of overlap between figures in the WT, QM-CtDGAT2a and QM-CtDGAT2b panels.
- Figure 5A: there are duplications between different yeast growth panels within the figure.
- Figure 5B: there is a duplication between the left plates for C18:3 and C20:4.
- Figure 6C: several yeast growth images appear to be derived from the same source image, involving panels ΔD2, ΔD4 and ΔD5.
- Figure 7A: There appear to be areas of overlap between the right panels for ΔD1(1–21) and ΔD5(481–571).

The concerns were brought to the attention of the authors, who provided replicate images of the same experimental sets but were not able to provide the images underlying the figures included in the article.

An institutional investigation conducted by The Indian Institute of Technology at Kharagpur confirmed the duplication of images and recommended the retraction of the article.

The preparation of the figures falls below the standard of publication; in light of these concerns and in line with the institutional recommendation, the authors and the editors retract this publication.

The authors sincerely apologize to the scientific community for the errors in the published article.
